# The predictive performance of short-linear motif features in the prediction of calmodulin-binding proteins

**DOI:** 10.1186/s12859-018-2378-9

**Published:** 2018-11-20

**Authors:** Yixun Li, Mina Maleki, Nicholas J. Carruthers, Paul M. Stemmer, Alioune Ngom, Luis Rueda

**Affiliations:** 1 School of Computer Science, University of Windsor, Windsor, Ontario Canada; 2 Inst. of Env. Health Sci., Wayne State University, Detroit, MI USA

## Abstract

**Background:**

The prediction of calmodulin-binding (CaM-binding) proteins plays a very important role in the fields of biology and biochemistry, because the calmodulin protein binds and regulates a multitude of protein targets affecting different cellular processes. Computational methods that can accurately identify CaM-binding proteins and CaM-binding domains would accelerate research in calcium signaling and calmodulin function. Short-linear motifs (SLiMs), on the other hand, have been effectively used as features for analyzing protein-protein interactions, though their properties have not been utilized in the prediction of CaM-binding proteins.

**Results:**

We propose a new method for the prediction of CaM-binding proteins based on both the total and average scores of known and new SLiMs in protein sequences using a new scoring method called sliding window scoring (SWS) as features for the prediction module. A dataset of 194 manually curated human CaM-binding proteins and 193 mitochondrial proteins have been obtained and used for testing the proposed model. The motif generation tool, Multiple EM for Motif Elucidation (MEME), has been used to obtain new motifs from each of the positive and negative datasets individually (the SM approach) and from the combined negative and positive datasets (the CM approach). Moreover, the wrapper criterion with random forest for feature selection (FS) has been applied followed by classification using different algorithms such as *k*-nearest neighbors (*k*-NN), support vector machines (SVM), naive Bayes (NB) and random forest (RF).

**Conclusions:**

Our proposed method shows very good prediction results and demonstrates how information contained in SLiMs is highly relevant in predicting CaM-binding proteins. Further, three new CaM-binding motifs have been computationally selected and biologically validated in this study, and which can be used for predicting CaM-binding proteins.

**Electronic supplementary material:**

The online version of this article (10.1186/s12859-018-2378-9) contains supplementary material, which is available to authorized users.

## Background

**Fig. 1:**
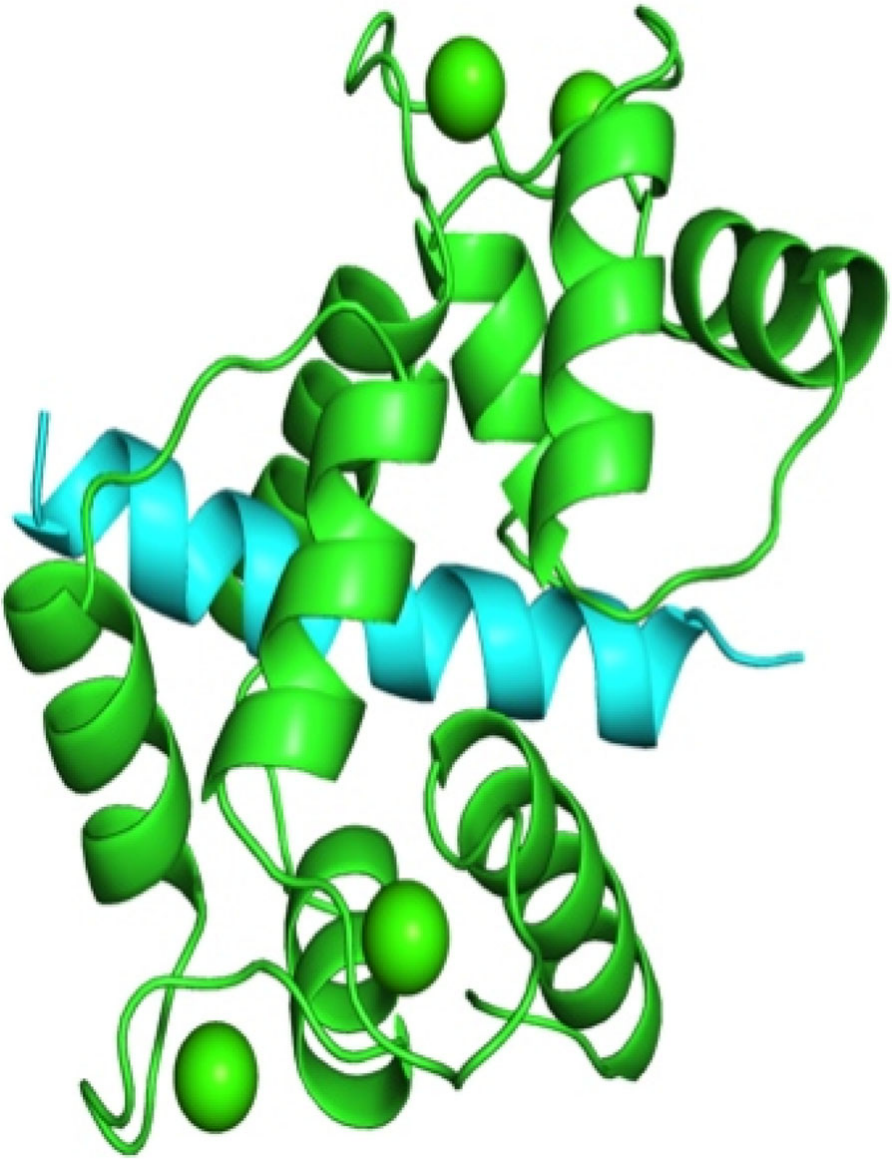
Quaternary structure of calcium-dependent interaction. Quaternary structure of CaM (green), visualized with ICM Browser[22], along with its interacting binding domain from calcineurin (blue)

Calmodulin (CaM) is a calcium-binding protein that is a major transducer of calcium signaling [1] and is a key signaling molecule for multicellular organisms. It has no enzymatic activity of its own but rather acts by binding to and altering the activity on a panel of cellular protein targets at a variety of motifs through binding mechanisms. Its targets are structurally and functionally diverse and participate in a wide range of physiological functions including immune response, muscle contraction and memory formation. Identifying CaM target proteins and CaM sites is an important and ongoing research problem because of the great diversity of conformations it uses in its target interactions. This diversity cannot be captured by a single amino acid sequence motif, but instead CaM-binding sites are commonly divided into four or more motif classes with different sequence characteristics [2]. Historically, CaM-binding sites have been categorized into motifs based on biochemical criteria [3]. Motifs can be either calcium-dependent or calcium-independent based on whether they interact with CaM at basal cellular calcium concentrations (independent) or require elevated calcium (dependent). The 1-10, 1-14 and 1-16 motifs are examples of calcium-dependent motifs and are named to indicate the positions of key hydrophobic residues involved in CaM interaction. Binding sites with the IQ motif are calcium-independent. Figure 1 is typical of a calcium-dependent interaction where the two halves of CaM bind to opposite sides of the target peptide (the four calcium molecules are green spheres). However, there is great diversity in how CaM can interact with its targets, making the prediction of CaM-binding motifs challenging. In addition, existing algorithms have difficulties in identifying novel CaM-binding proteins. For example, the Hidden Markov Model prediction tool in the Calmodulin Target Database [2] is limited to the classic CaM-binding motifs and has no power to identify novel ones.

On the other hand, short-linear motifs (SLiMs), patterns of 3 to 10 amino acids in intrinsically disordered regions of protein sequences, can encode functional aspects of proteins and bind to important domains [4]. They also help regulate many cellular processes, by being interaction sites for other SLiMs in proteins. SLiM-mediated interactions are often transient interactions or utilize additional interaction domains to co-operatively produce stable complexes. Therefore, prediction and analysis of CaM-binding proteins using SLiM profiles has the potential to develop better models for calcium-regulated cellular processes such as modulation and regulation of proliferation and apoptosis [5].

Recent studies have focused on the discovery of new SLiMs for the prediction of protein interactions [6–8]. Some commonly used SLiM tools are SLiMFinder [9], SLiMSearch [10], Minimotif Miner (MnM) [11], and MEME (Multiple EM for Motif Elucidation) [12]. MEME can discover SLiMs through an unsupervised approach and turns out to be a very efficient and successful algorithm for discovering new SLiMs with different numbers of occurrences in a set of protein sequences. It discovers motifs by optimizing the statistical parameters of the model using the Expectation Maximization (EM) algorithm, and a statistical sequence model to determine the positions and the width of the motif sites in the sequences [13].

In one of our recent works [14], a computational model was proposed for prediction and analysis of CaM-binding proteins using SLiM profiles. We used new SLiMs derived from MEME as features for prediction. Two different approaches were used to discover new motifs using MEME: (a) find SLiMs from each of the positive and negative datasets separately (SM) and (b) find SLiMs from the combined positive and negative datasets (CM). For each protein and for each SLiM, we scored the SLiM using a new scoring function, the Sliding Window Scoring (SWS), which is based on the number of sites containing the SLiM in the protein. The experimental results indicated that the classification using the SLiMs obtained from CM generally achieve better performance.

This paper is an extension of the work presented in [14] by employing known CaM-binding motifs for prediction of CaM-binding proteins and comparing the prediction results with the new discovered motifs from MEME. Predictions of CaM-binding proteins had been performed using *k*-nearest neighbors (*k*-NN), support vector machines (SVM), Naive Bayes (NB) and random forest (RF) classifiers available in the Waikato Environment for Knowledge Analysis (WEKA). The experimental results confirm that the new discovered motifs are relevant and crucial to predict CaM-binding proteins. Moreover, it has been demonstrated that prediction results are improved by applying feature selection approaches and identifying more relevant and discriminative features, while removing redundant and noisy ones for the most subsets of features. Furthermore, biological analysis performed on three computationally selected motifs (SLiMs #2, #43, and #52) have confirmed certain structural characteristics and properties which allows these three motifs to discriminate CaM-binding proteins.

## Methods

**Fig. 2:**
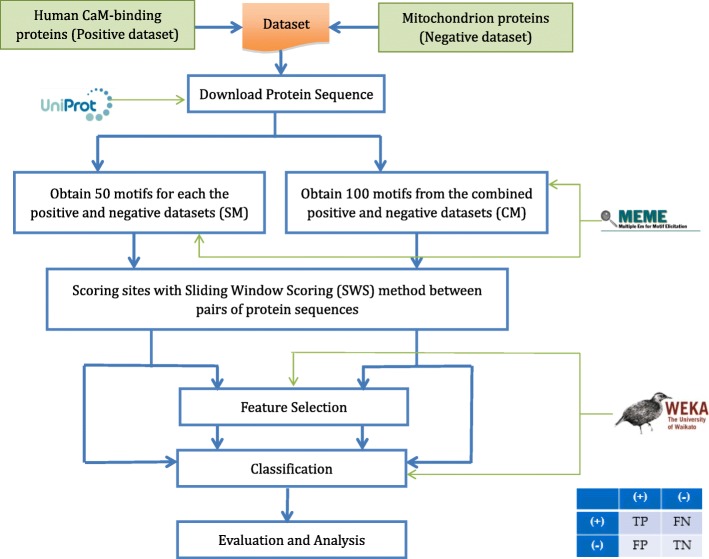
Diagram of the proposed computational model. After extracting the FASTA sequences of all the proteins in the dataset and scoring the motifs (features), using one of the SWS methods and selecting the best ones, predictions have been performed using a classification method

Our proposed model to predict CaM-binding proteins is illustrated in the Fig. 2. First, the FASTA sequences of all the CaM and non CaM-binding proteins in the dataset have been downloaded from the Uniprot database [15]. Second, we use MEME to extract new SLiMs to be used as predictive features. Third, given each protein we scored each SLiM feature using our novel SWS scoring methods (to be introduced in this section) and then applied feature selection methods to obtain subsets of the most relevant and distinguishing predictive features. Finally, we applied SVM, RF, and NB classifiers on the CM and SM datasets, by using only the selected SLiM features. Detailed discussion of the dataset, scoring methods, feature selection methods and classifiers is provided in this section.

### Datasets

Our manually curated dataset contains 194 human CaM-binding proteins collected from the Calmodulin Target Database [2] used as the positive dataset, and 193 mitochondrial proteins (mt-proteins) obtained from the Uniprot database as the negative dataset. Mt-proteins were chosen as a negative dataset because no major biochemical function has been demonstrated for CaM in the mitochondria suggesting that the number of CaM-interacting proteins that are localized in the mitochondria is small relative to other sub-cellular locations. Gene Ontology (GO) cellular component annotations were used to identify mt-proteins so that our negative dataset includes proteins encoded in both the mitochondrial and nuclear genomes. To construct the list, we downloaded 7433 proteins that were under the cellular component term “Mitochondrion” (GO:0005739). After filtering out non-reviewed proteins and any proteins with “Golgi” or “Nucleus” annotations, 886 proteins were obtained, which are strictly mitochondrial as far as GO annotations are concerned. From those remaining mt-proteins, 193 proteins, which contain a few if any CaM-binding regions, were selected manually as the negative dataset, yielding a balanced dataset. The final dataset used in this study is included in Additional file 1.

### Scoring the sites

In this paper, two different scoring methods are proposed. The SWS_PPM method is used to score the newly discovered motifs from MEME, while the SWS_RE method is mainly employed to score the previously known CaM-binding motifs using regular expressions.

#### The SWS_PPM method

Once the SLiM sets are obtained, MEME outputs files that contain the patterns for the SLiMs, sites found in the protein sequences and their positions, and the probability matrix of the features of each SLiM.

**Fig. 3:**
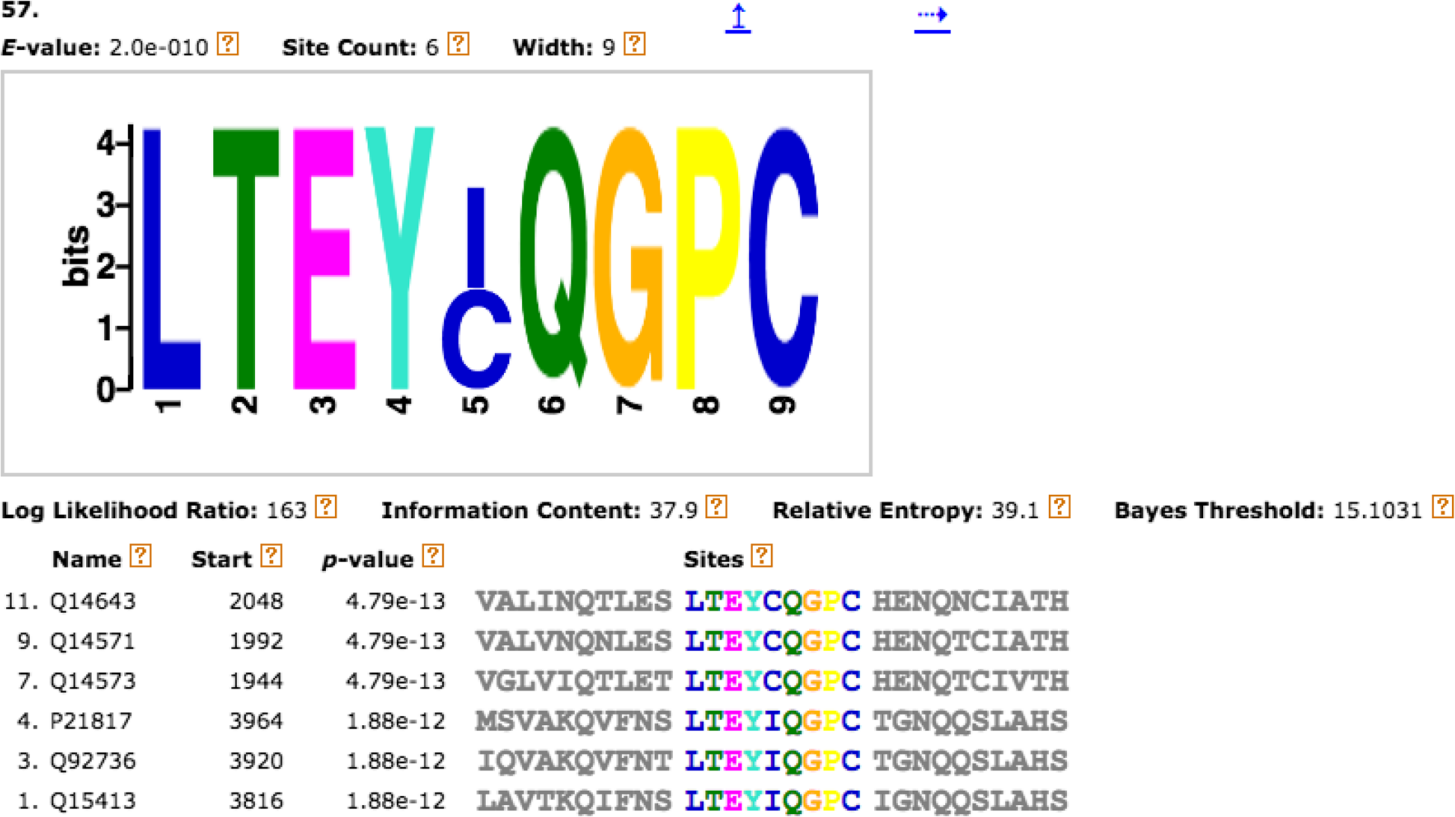
SLiM #57 found by CM. SLiM #57 found in the dataset obtained by CM output by MEME with the sites found in the sequences and the corresponding protein names

MEME outputs its results as interactive HTML, XML and text files. The patterns of SLiMs as well as the sequences that contain the sites of SLiMs are in the HTML file. The regular expressions of SLiMs and the weights of different amino acids in each SLiM are in the XML and text files. The Position-Specific Probability Matrix (PSPM) of each motif can be found in the text file. Figure 3 shows SLiM #57 found in the dataset obtained by CM output by MEME with the sites found in the sequences and the corresponding protein names. Table 1 shows the PSPM of this SLiM. The columns represent the 20 amino acids, while the rows correspond to the positions in the corresponding site; each entry value in the matrix is the probability that a given amino-acid appears at that particular position in the site. From Fig. 3, we observe that the regular expression of this SLiM is “LTEY[IC]QGPC”, and the sites of this SLiM appear in the proteins: Q14643, Q14571, Q14573, P21827, Q92736, and Q15413. Furthermore, the site can be found either as LTEYIQGPC or LTEYCQGPC in those proteins. From Table 1 we observe that in the first position, the probability score of amino acid L is 1.0 and others are 0; hence, L is the only amino acid in this position. The same logic applies for the other positions except for position No. 5 where the probability scores of amino acids C and I are both 0.5; hence, either can appear at the 5th position in this SLiM. They have the same size in the pattern shown in Fig. 3. The sizes of different amino acids in the same position depend on their probability scores, the greater the score is the longer the site is.

**Table 1:** Position-specific probability matrix of SLiM #57

Position	A	C	D	E	F	G	H	I	K	L	M	N	P	Q	R	S	T	V	W	Y
1	0	0	0	0	0	0	0	0	0	1.0	0	0	0	0	0	0	0	0	0	0
2	0	0	0	0	0	0	0	0	0	0	0	0	0	0	0	0	1.0	0	0	0
3	0	0	0	1.0	0	0	0	0	0	0	0	0	0	0	0	0	0	0	0	0
4	0	0	0	0	0	0	0	0	0	0	0	0	0	0	0	0	0	0	0	1.0
5	0	0.5	0	0	0	0	0	0.5	0	0	0	0	0	0	0	0	0	0	0	0
6	0	0	0	0	0	0	0	0	0	0	0	0	0	1.0	0	0	0	0	0	0
7	0	0	0	0	0	1.0	0	0	0	0	0	0	0	0	0	0	0	0	0	0
8	0	0	0	0	0	0	0	0	0	0	0	0	1.0	0	0	0	0	0	0	0
9	0	1.0	0	0	0	0	0	0	0	0	0	0	0	0	0	0	0	0	0	0

**Fig. 4:**
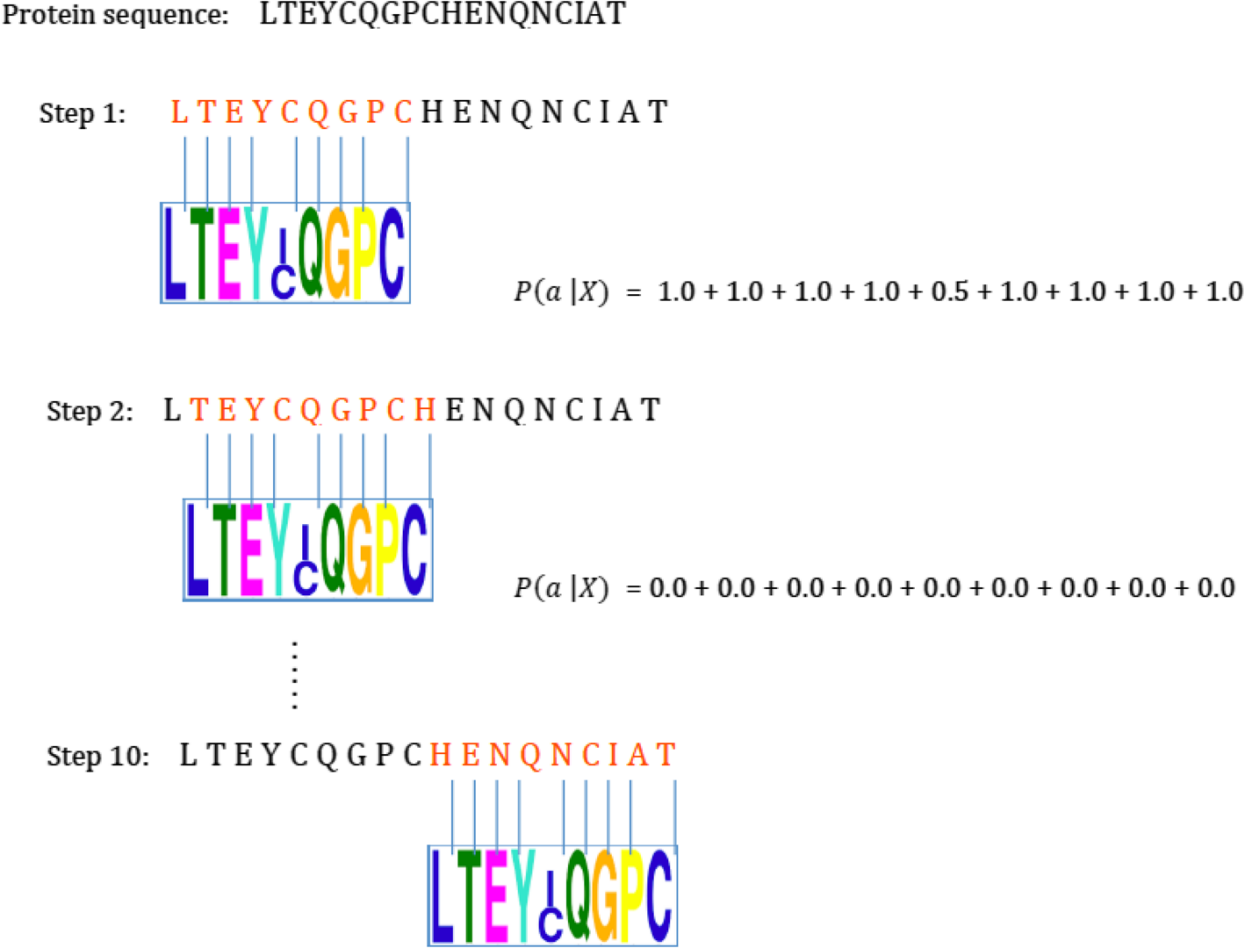
Example of the SWS_PPM method based on SLiM #57 along with its PSPM. An example of SWS_PPM based on SLiM #57 along with its position-specific probability matrix. We considered every possible sub-sequence in a sequence as a potential site for a motif of the training set. Each time we move the SLiM window to score the site using the scores of amino acids based on its PSPM. At the end of each sequence we add up all scores of sites together as the score of the SLiM on this protein sequence

We did not consider only the sites in the sequences found by MEME. In contrast, we considered every possible sub-sequence (*l-mer*) in a sequence as a potential site for a motif of the training set. Each sequence is divided into overlapping *l-mers*. We designed the SWS_PPM probability matrix (PSPM) representation of a motif. Figure 4 shows an example of SWS_PPM based on SLiM #57 along with its position-specific probability matrix. Let us consider *l*-*mera* in a protein sequence *A* of length *L*. We divide the sequence into all possible overlapping *l*-*mers* of length *W* (i.e., potential sites), where *l*=*W* is the length of each SLiM, delivering a total of {*L*−*W*+1}*l-mers*. Then, Eq. (1) is used to calculate the information contained in *l-mera*, given a PSPM *X* of a SLiM *m* of length *W*:

1 $$ P(a |\ X) = \sum_{i=1}^{W}P(a_{i}),  $$

where *P*(*a*_*i*_) is the probability of the amino acid at position *i* in *a*. Only potential sites whose values *P*(*a*|*X*)≥60*%* are considered true sites and thus retained. Equation (2) adds up the scores of all the true sites as the score of SLiM *m* given protein sequence *A* of length *L*, as follows:

2 $$ P(m |\ A) = \sum_{i=1}^{L - W + 1}P(a |\ X).  $$

Equation (2) implies that the more likely that *a* is a site, the larger the information content is. Thus, in order to erase this effect, we also divide the total information content by the number of true sites (given SLiM *m*) found in the protein sequence, *N* ≤*L*−*W*+1, since we removed all potential sites with values *P*(*a*|*X*)≥60*%*:

3 $$ \hat{P}(m |\ A) =\frac{1}{N} \times {\sum_{i=1}^{L - W + 1}P(a |\ X)}.  $$

For each protein *p*_*i*_, we compute the *P*(*m*| *A*) and $\hat {P}(m |\ A)$ values for each SLiM *m* obtained from both SM and CM datasets. Given the set of *n* SLiMs, *m*_1_,*m*_2_, …, *m*_*n*_, we transform each protein *A*_*i*_ into two feature vectors *S*_*i*_=(*s*_*i*1_,*s*_*i*2_, …, *s*_*in*_) and *T*_*i*_=(*t*_*i*1_,*t*_*i*2_, …, *t*_*in*_); where, *s*_*ij*_=*P*(*m*_*j*_|*A*_*i*_) and where, $t_{ij} = \hat {P}(m_{j} | A_{i})$, respectively, given protein *A*_*i*_. The corresponding matrices that we obtain are called the *S*-score matrix and *T*-score matrix. This transformation is applied to each protein in the negative data and the positive data in the training set, given all the SLiMs obtained from both the SM and CM approaches.

#### The SWS_RE method

**Fig. 5:**
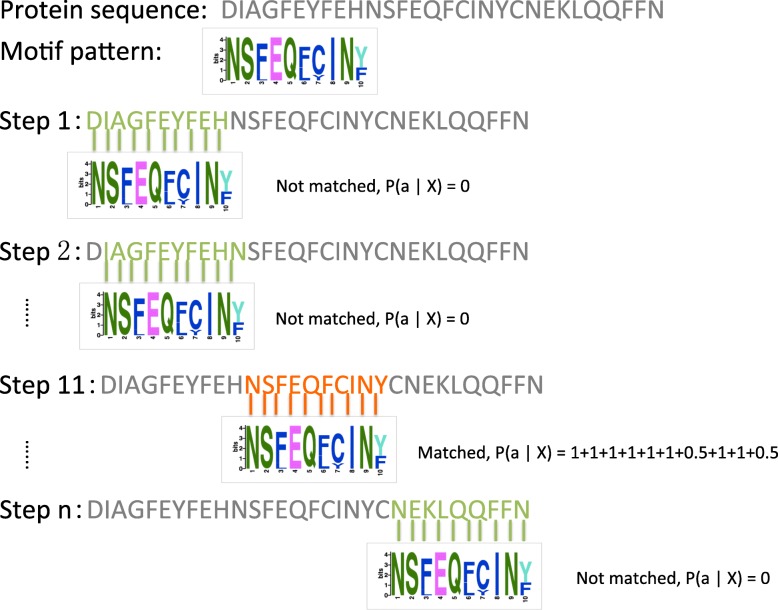
Example of the SWS_RE method based on SLiM # 1. An example of SWS_RE based on SLiM #1 found in the dataset obtained by CM output by MEME. We considered every possible sub-sequence in a sequence as a potential site for a motif of the training set. We then move the SLiM window and use regular expression to check if the SLiM pattern matches each site. Then we do not consider the potential site as a site; otherwise, we score the site. At the end of each sequence we add up all scores of sites together as the score of the SLiM for this protein sequence

Similar to the SWS_PPM scoring method, we consider every possible *l*-*mer* in a sequence as a potential site for a motif of the training set, and score each *l*-*mer* using a new scoring method, called SWS_RE, which is based on the regular expression representation of the motif. Figure 5 shows an example of the SWS_RE scoring process using SLiM #1 along with its score. Let us consider *l*-*mera* in a protein sequence *A* of length *L*. We divide the sequence into all possible overlapping *l*-*mers* of length *W*, where *l*=*W* is the length of each SLiM, which gives a total of {*L*−*W*+1}*l*-*mers*. We then use the SLiM’s regular expression to check if the SLiM pattern matches each *l*-*mer*. If the SLiM pattern does not match a given*l*-*mer*, then the *l*-*mer* is not considered to be a true site, otherwise *l*-*mer* is a true site. When the *l*-*mer* is a true site, we use Eq. (1) to calculate the information contained in *l*-*mera*, given the regular expression pattern X of a SLiM *m* of size *l*=*W*, and a SLiM *m* of length *W*.

Unlike in the SWS_PPM method, here, we define the value of *P*(*a*_*i*_) using regular expression X as follows: the score of position *i* is 1/(number of amino-acids at position *i*). For example, given a SLiM pattern “[IL]QxW” of length *W*=4, if the *l*-*mer* is a true site that matches this SLiM, then the first amino-acid *a*_1_ can only be “I” or “L”, and hence, *P*(*a*_1_)=1/2=0.5. *P*(*a*_2_), *P*(*a*_3_), and *P*(*a*_4_) will be set to one each. Once the scores for all possible *l*-*mers* in protein sequence *A* are obtained, we use Eq. (2) to add up all the scores of the *l*-*mers* as the score of SLiM *m* for sequence *A*.

Then, we calculate *P*(*m*| *A*) for all the SLiMs obtained from CM for each protein sequence, and transform each protein sequence *A*_*i*_ into a feature vector *S*_*i*_=(*s*_*i*1_,*s*_*i*2_, …, *s*_*in*_); where, *s*_*i*_*j*=*P*(*m*_*j*_|*A*_*i*_) given *A*_*i*_.

### Classification

There are a variety of classification methods, of which SVM, RF and *k*-NN and NB are four of the most well-known ones, and which are used in this study.

#### Support vector machine

SVMs are well known machine learning techniques used for classification and regression. The aim of the SVM is to find the hyperplane that ideally separates the feature space into two regions (classes). As this kind of hyperplane is not unique, the SVM chooses the hyperplane that gives the maximum margin from that hyperplane to the support vectors. The classification by using the SVM is usually inaccurate when using a linear classifier, because in general, the data are not linearly separable. Thus, the use of kernels is crucial in implicitly mapping the data onto a higher dimensional space in which the classification is more accurate. The effectiveness of the SVM depends on the selection of the kernel, the selection parameters and the soft margin [16]. There are a number of different kernels that can be used in SVMs such as polynomial, radial basis function (RBF), sigmoid, and many others. In addition, sequential minimal optimization (SMO) is a fast SVM learning algorithm that has been widely applied in the training phase of a SVM classifier as one possible way to solve the underlying quadratic optimization problem. In this work, the SMO module of WEKA with a normalized polynomial kernel, default parameter settings, and 3-fold cross-validation is used to perform classification via the SVM [17].

#### Random forest

RF is a classifier that uses an ensemble (i.e., forest) of decision tree predictors such that each tree depends on the values of a random vector sampled independently and with the same distribution for all trees in the forest. RF achieves excellent predictive performance among current classification algorithms. It also has an effective method for estimating missing data and maintains accuracy when a large proportion of the data is missing [18]. In this study, the RandomForest module of WEKA with default parameters is used [17].

#### *k*-Nearest neighbor

The *k*-NN rule is among the simplest of all machine learning methods and is a type of instance-based/lazy learning method. To find the class of a test sample, first, the distances between the test sample and each training sample should be calculated and sorted. Then, the most frequent class label in the first *k* training samples (nearest neighbors) is assigned to the test sample. One of the main challenges of this method is to determine the best number of neighbors. In this study, the IBK module of WEKA with Euclidean distance is used [17].

#### Naive Bayes

One of the simplest probabilistic classifiers is NB. Assuming independence of the features, the class of each test sample can be found by applying Bayes’ theorem. The basic mechanism of NB is rather simple. The reader is referred to [19] for more details. In this study, the NaiveBayes module of WEKA with default parameters is used [17].

### Feature selection

Applying feature selection methods before running a classifier is important in order to reduce the dimensionality of the data by discarding redundant and/or irrelevant features, and, thus, reducing the prediction time, while improving the classification performance.

In this paper, we applied the wrapper approach with RF for feature selection followed by classification using different algorithms. Wrapper methods embed the model hypothesis search within the feature subset search. In this context, a search procedure in the space of possible feature subsets is defined, and various subsets of features are generated and evaluated. The evaluation of a specific subset of features is obtained by training and testing a specific classification model, rendering this approach tailored to a specific classification algorithm [20].

Also, feature selection via the Chi square test is another, very commonly used method [21]. This method evaluates the relevance of a feature with respect to a class by computing the value of the Chi square statistic. In this study, the ChiSquaredAttributeEval module of WEKA is used to obtain the scored feature vector.

## Results and discussion

To test our proposed method and perform an in-depth analysis of the strength of SLiMs as the prediction properties, four different classification methods including SVM, *k*-NN, RF and NB, and different feature selection methods including Chi2 and the wrapper RF method have been used on our datasets using WEKA ver. 3.7.11 [17].

The performances of the prediction methods are compared in terms of their areas under the receiving operating characteristics (ROC) curve, accuracies, and Matthews correlation coefficient (MCC) which are computed as follows:

4 $$\begin{array}{*{20}l} Accuracy =\frac{TP+TN}{TP+FP+TN+FN}, \end{array} $$

5 $$\begin{array}{*{20}l} MCC =\frac{TP*TN - FP*FN}{\sqrt{(TP+FP)(TP+FN)(TN+FP)(TN+FN)}}, \end{array} $$

where *TP* and *TN* are the total numbers of true positive (CaM-binding proteins) and true negative (mt-proteins), respectively, and *N*=*TP*+*FP*+*TN*+*FN* is the total number of proteins in the dataset.

### Analysis of prediction properties using cross validation approach

The classification results for the score matrices with SLiMs obtained from the SM and CM datasets using SWS_PPM method following 3-fold cross validation are shown in Tables 2 and 3, respectively.

**Table 2:** Classification results for the score matrices with SLiMs obtained from SM using 3-fold cross validation

Dataset for classification	# features	Classifier	Accuracy (%)	MCC	ROC Area
*S* score matrix		SVM-Polynomial	72.6	0.45	0.73
	100	RF	73.1	0.46	0.81
		*k*-NN (*k*=1)	80.6	0.61	0.81
*T* score matrix		SVM-Polynomial	55.0	0.11	0.55
	100	RF	68.5	0.38	0.77
		*k*-NN (*k*=1)	59.7	0.27	0.60

**Table 3:** Classification results for the score matrices with SLiMs obtained from CM using 3-fold cross validation

Dataset for classification	# features	Classifier	Accuracy (%)	MCC	ROC Area
*S* score matrix		SVM-Polynomial (*c*=1,*g*=0)	72.6	0.45	0.73
	100	RF	74.7	0.49	0.74
		*k*-NN (*k*=1)	78.3	0.57	0.78
*T* score matrix		SVM-Polynomial (*c*=1,*g*=0)	57.6	0.21	0.58
	100	RF	69.3	0.40	0.77
		*k*-NN (*k*=1)	58.1	0.26	0.58

From the tables, it is noticeable that (a) *k*-NN on the *S* score matrix yields the highest classification accuracy of 80.6 and 78.3% for the SLiMs obtained from SM and CM, respectively; (b) the *S* score matrix is a better subset of features than the *T* score matrix for both SM and CM; (c) using the motifs from the combined negative and positive datasets (CM dataset) yielded better results than the motifs obtained from each of the positive and negative datasets individual (SM dataset) in most of the experiments.

### Analysis of prediction properties using the holdout approach

**Fig. 6:**
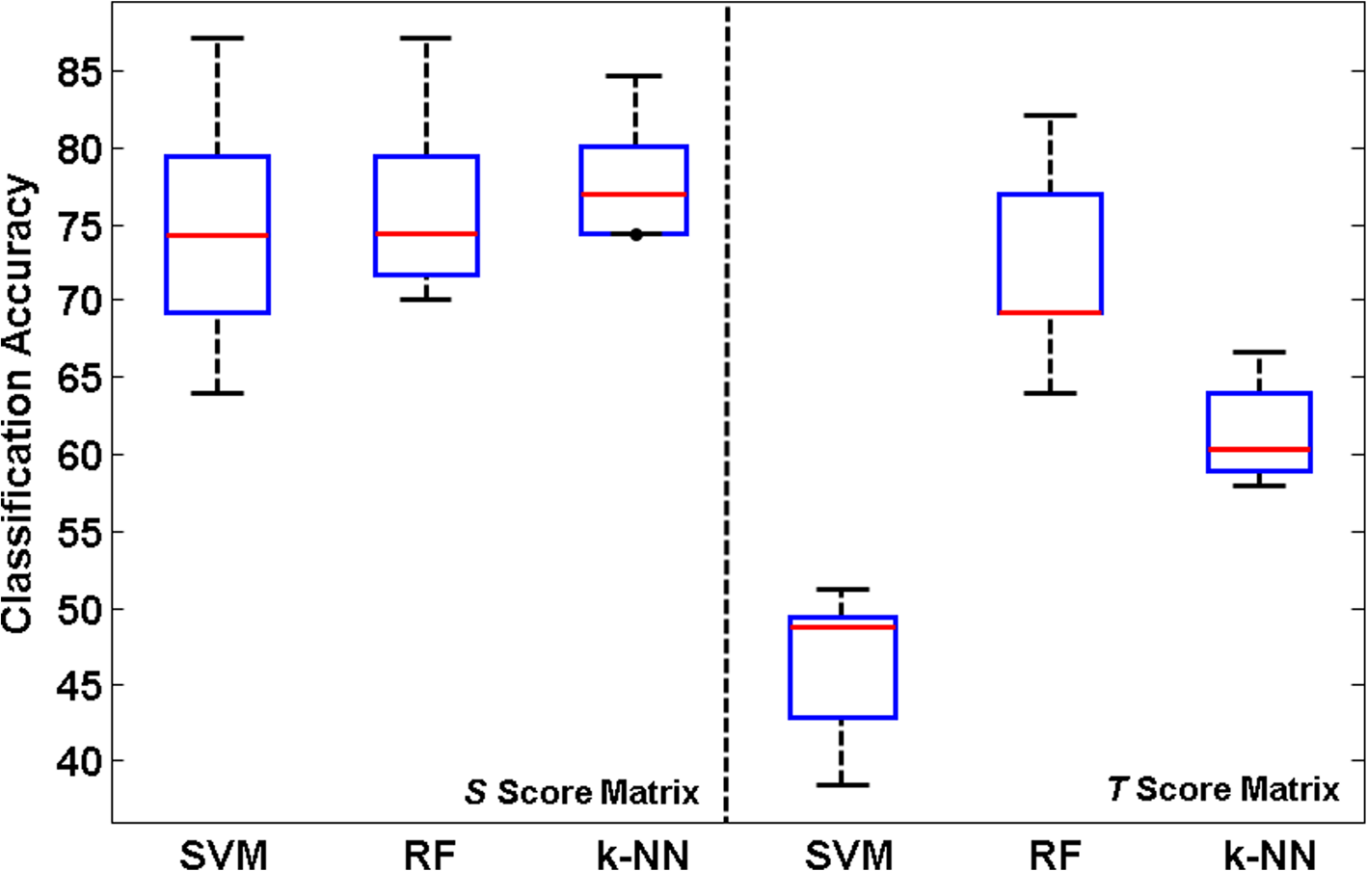
Box plot obtained from SM. Classification results for the score matrices with SLiMs obtained from SM using the holdout approach

**Fig. 7:**
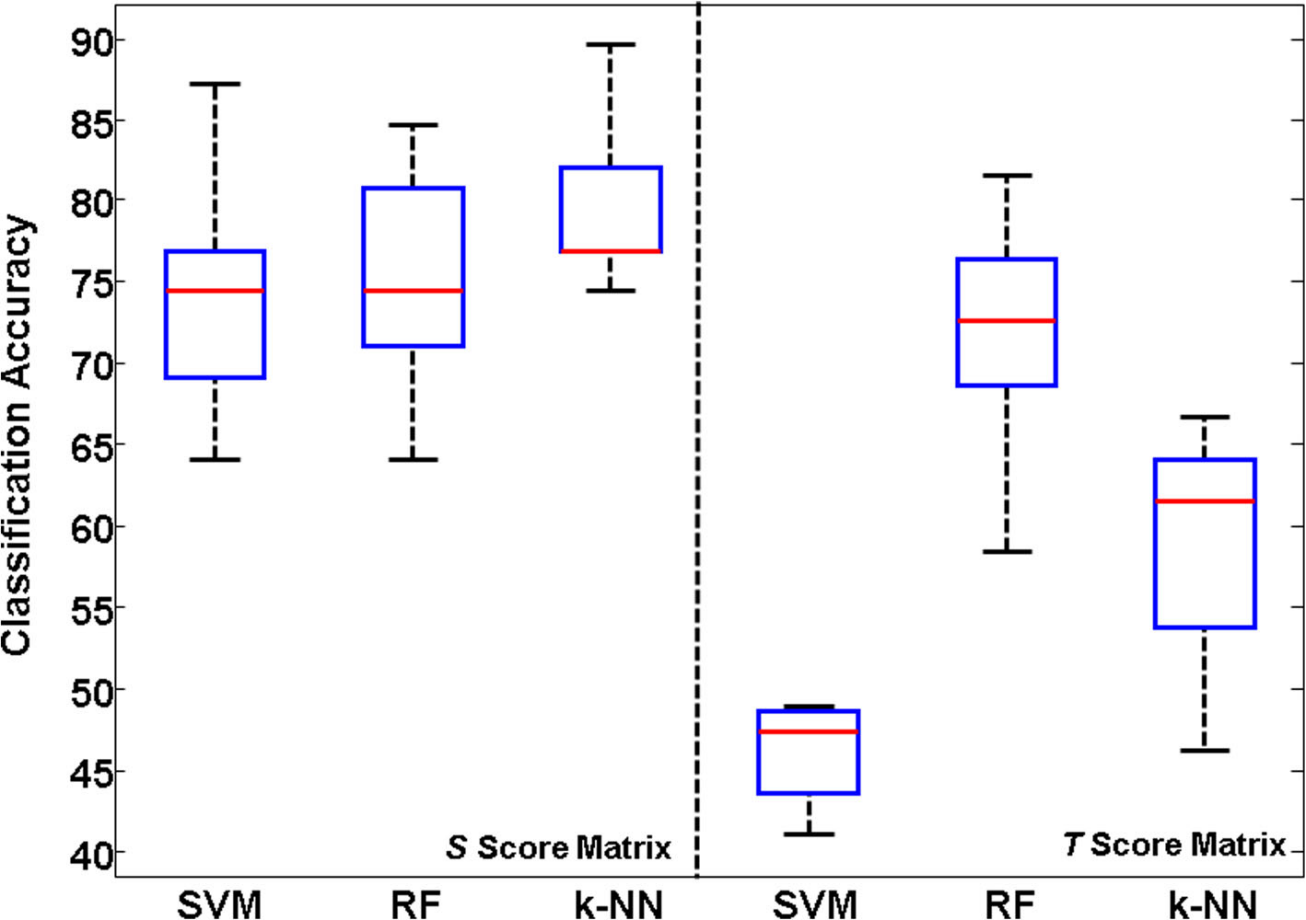
Box plot obtained from CM. Classification results for the score matrices with SLiMs obtained from CM using the holdout approach

Besides the cross-validation approach, a classifier can also be evaluated using the holdout approach (percentage split), in which a certain percentage of the dataset is used to train and the rest used for testing. As another experiment, independent random seeds from 1 to 10 in WEKA have been used to produce a percentage split of the score matrices with SLiMs obtained from the SM and CM datasets into 90% for training and 10% for the test set. After employing SVM, RF and *k*-NN classifiers on each split, the median, minimum, maximum, and first and third quartile values have been calculated and visualized on the box plots (Figs. 6-7). Similar to the results presented in Tables 2 and 3, it is clear from the box plots that the *S* score matrix is a better subset of features than the *T* score matrix for both SM and CM.

### Analysis of feature selection

As another experiment, the wrapper approach with RF was applied to score and rank the features, while SVM, RF and *k*-NN were employed for classification. The performances of the classifiers using different numbers of selected features for *S* and *T* score matrices obtained from SM and CM, are shown in Tables 4 and 5, respectively. For the SLiMs obtained from SM, the subset obtained using feature selection contains seven features for the *S* score matrix, and nine features for the *T* score matrix. As for the SLiMs obtained from CM, the subset obtained from FS contains eleven features for the *S* score matrix, and seven features for the *T* score matrix. Similarly, from Tables 4 and 5, it is clear that RF on the *S* score matrix yield the highest classification accuracy of 77.8% and 80.1% for the SLiMs obtained from SM and CM, respectively. Also, it is observable that the classification using the SLiMs obtained from CM yields better performance than using the SLiMs obtained from SM.

**Table 4:** Classification results for the score matrices with SLiMs obtained from SM using FS

Dataset for classification	# features	Classifier	Accuracy (%)	MCC	ROC Area
*S* score matrix		SVM-Polynomial (*c*=1,*g*=0)	66.1	0.33	0.66
	7	RF	77.8	0.56	0.83
		*k*-NN (*k*=1)	77.0	0.54	0.77
*T* score matrix		SVM-Polynomial (*c*=1,*g*=0)	53.0	0.09	0.53
	9	RF	69.3	0.38	0.75
		*k*-NN (*k*=1)	66.4	0.33	0.66

**Table 5:** Classification results for the score matrices with SLiMs obtained from CM using FS

Dataset for classification	# features	Classifier	Accuracy (%)	MCC	ROC Area
*S* score matrix		SVM-Polynomial (*c*=1,*g*=0)	62.0	0.24	0.62
	11	RF	80.1	0.60	0.85
		*k*-NN (*k*=1)	78.6	0.57	0.79
*T* score matrix		SVM-Polynomial (*c*=1,*g*=0)	60.2	0.21	0.60
	9	RF	70.5	0.415	0.80
		*k*-NN (*k*=1)	68.7	0.38	0.69

Moreover, comparing the classification results obtained by using the feature selection method (Tables 4 and 5) with no feature selection (Tables 2 and 3) demonstrate the strength of the feature selection method in selecting more powerful and discriminating features for classification for the most subsets of features. However, the maximum decrease of 6% on the classification performance is still acceptable because the classification performed faster using a smaller number of features.

### Comparison with the previously known motifs

In this part, the classification results of CaM-binding proteins using the motifs discovered in this study have been compared with other studies. As mentioned earlier, CaM-binding sites have been previously categorized into motifs based on biochemical criteria in [3]. In this experiment, the classification results of SVM, NB and RF using 14 previously-known canonical CaM-binding motifs (Table 6) and 100 new discovered SLiMs from MEME following 10-fold cross validation have been compared. The results are shown in Table 7. To score the features, the SWS_RE method has been employed because there is no way to find the PSPM table of the known motifs.

**Table 6:** Canonical CaM-binding motifs obtained from [3]

Motifs	Sequence
1-10	[FILVW]xxxxxxxx[FILVW]
1-5-10	[FILVW]xxx[FAILVW]xxxx[FILVW]
Basic 1-5-10	[RK][RK][RK][FAILVW]xxx[FILVW]xxxx[FILVW]
1-12	[FILVW]xxxxxxxxxx[FILVW]
1-14	[FILVW]xxxxxxxxxxxx[FILVW]
1-8-14	[FILVW]xxxxxx[FAILVW]xxxxx[FILVW]
1-5-8-14	[FILVW]xxx[FAILVW]xx[FAILVW]xxxxx[FILVW]
Basic 1-8-14	[RK][RK][RK][FILVW]xxxxxx[FAILVW]xxxxx[FILVW]
1-16	[FILVW]xxxxxxxxxxxxxx[FILVW]
IQ	[FILV]Qxxx[RK]Gxxx[RK]xx[FILVWY]
IQ-like	[FILV]Qxxx[RK]xxxxxxxx
IQ-2A	[IVL]QxxxRxxxx[VL][KR]xW
IQ-2B	[IL]QxxCxxxxKxRxW
IQ unconventional	[IVL]QxxxRxxxx[RK]xx[FILVWY]

**Table 7:** Classification results using known and new CaM-binding motifs, 10-fold cross validation and SWS_RE scoring method

Dataset for classification	# features	Classifier	Accuracy (%)
Known SLiMs		SVM	49.09
	14	RF	70.80
		NB	70.03
New SLiMs		SVM	71.58
	100	RF	71.32
		NB	71.06

From Table 7, it is clear that SVM yields the highest classification accuracy of 71.58% for the SLiMs obtained from MEME, while the best accuracy using known motifs is 70.80% using the RF classifier. Although the reported accuracies using the new motifs obtained from MEME are not much higher than using the 14 previously known CaM-binding motifs, it is still acceptable and valuable because it leads to newly discovered CaM-binding motifs.

### Biological analysis on the selected SLiMs

As another experiment, ten new motifs were selected from the ranked motifs produced by different feature selection methods including Chi square and wrapper methods. Then, only three of these ten motifs, SLiMs #2, #43 and #52, were finally selected by employing the recursive backward elimination technique.

The classification results of NB, RF and *k*-NN with 10-fold cross validation using these three SLiMs as well as the original 100 discovered new motifs are shown in Table 8. In this experiment, the SWS_PPM method is used for scoring the features. From the table, it is clear that the accuracy of *k*-NN increased from 74.45 to 77.77% and NB from 70.80 to 74.42% by using only the three selected motifs of 2, 43 and 52 instead of the original 100 motifs. The computational results indicate that SLiMs #2, #43 and #52 are the most relevant and discriminative motifs for prediction of CaM-binding proteins.

**Table 8:** Classification results for the score matrices with 3 and 100 SLiMs obtained from CM using the SWS_PPM scoring method

Classification method	3 Features	100 features
NB	74.72	70.8
RF	74.42	74.7
*k*-NN (*k*=3)	77.77	74.45

**Fig. 8:**
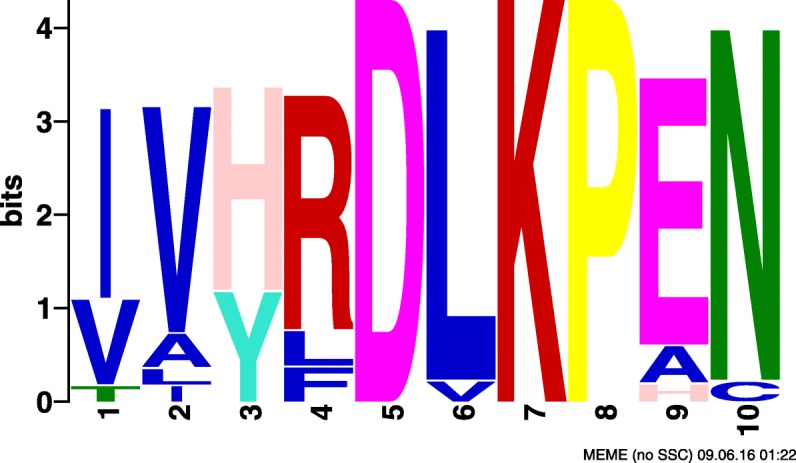
SLiM #2 found by CM. SLiM #2 found in the dataset obtained by CM output by MEME

**Fig. 9:**
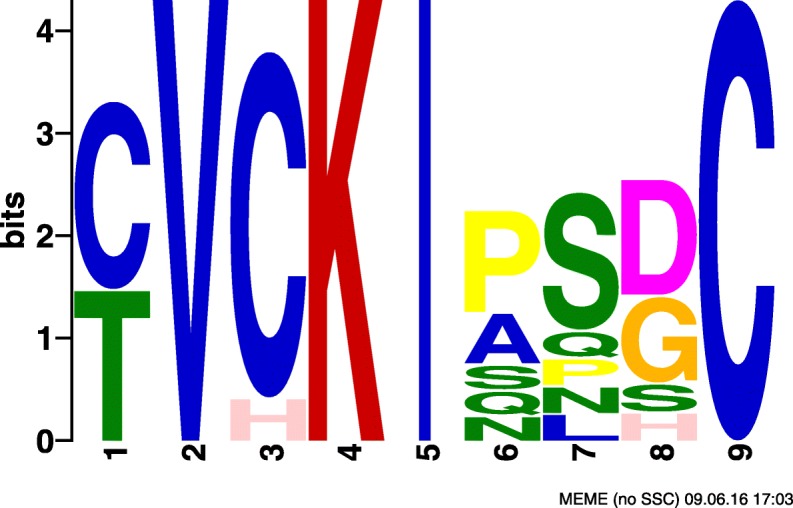
SLiM #43 found by CM. SLiM #43 found in the dataset obtained by CM output by MEME

**Fig. 10:**
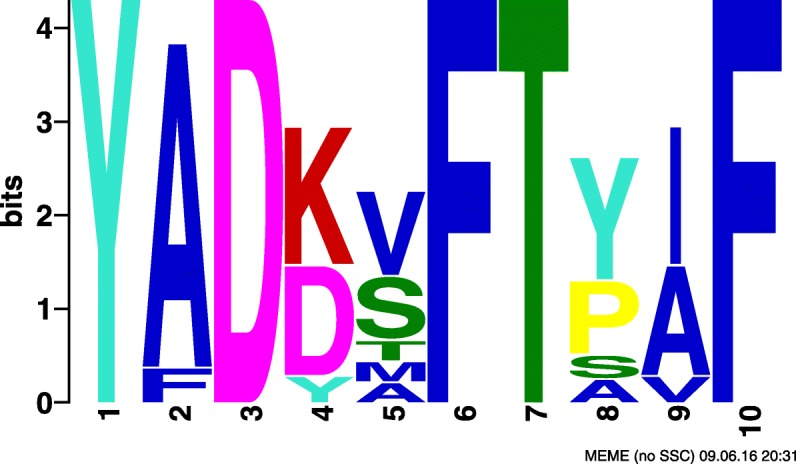
SLiM #52 found by CM. SLiM #52 found in the dataset obtained by CM output by MEME

Motif amino acid composition for these three SLiMs were examined considering motif positions that have more than 50% occupancy for a single amino acid or amino acid class. Canonical calcium dependent CaM-binding motifs are rich in basic and hydrophobic amino acids. Although not compulsory for interaction with calmodulin, motifs that are rich in basic and hydrophobic amino acids are of interest as they are in accordance with most literature regarding calcium dependent calmodulin interactions. SLiMs #2 and #43 each have a combination of basic and hydrophobic residues that are typical of calcium dependent CaM-binding domains: three basic and three hydrophobic positions for SLiM #2 and one basic and two hydrophobic positions for SLiM #43 (Table 9, Figs. 8, 9 and 10). SLiM #52 has no basic positions but four hydrophobic ones. Motifs that occur in diverse proteins are also biologically interesting as they may represent a general feature of CaM-binding proteins rather than a feature specific to a protein subset. SLiM #2 occurs in a set of 17 Kinases while SLiMs #43 and #52 occur in 9 and 10 proteins respectively with no obvious unifying protein class. Since these motifs were relevant in classifying CaM-binding proteins, they may represent novel CaM-binding or interaction sites or motifs that are otherwise characteristic of CaM-binding proteins.

**Table 9:** Biological analysis of selected motifs, where^a^ indicates the number of positions with at least 50% occupancy for the amino acid type

Motif #	Protein class	# Proteins	Basic residues^a^	Hydrophobic residues^a^
2	Kinases	10	3	3
43	N/A	9	1	2
52	N/A	17	0	4

## Conclusions

We propose a method for prediction of calmodulin-binding proteins using short-linear motifs. Our method shows very good results and demonstrates that information contained in SLiMs is highly relevant for accurate prediction of CaM-binding proteins and differentiate them from mitochondrial proteins. The SWS method is useful for scoring the sites and obtaining the datasets for classification. Most of the classifiers perform better on the total scores without dividing by the frequency of the SLiMs. The classification experiments yield good results on the datasets with SLiMs obtained from both of the SM and CM approaches. The 80.6% classification accuracy using *k*-NN as the classifier on the total scores obtained from SM is the highest accuracy among all of the experiments. Moreover, the performance of the classifiers improved for most subsets of features by using fewer informative features (SLiMs) selected by the wrapper approach with RF. Also, our biological analysis confirms that selected SLiMs #2, #43 and #52 may represent novel CaM-binding or interaction sites or motifs that are otherwise characteristic of CaM-binding proteins.

Further investigation will help understand the functional significance of these three selected motifs obtained by MEME to calmodulin-target interactions. Also, possible extension to this work is to investigate the SWS approach on prediction of other types of protein-protein interactions. Another extension to this work is to combine structural and SLiM data in order to achieve a better insight of the location of the motifs on the interface, role on the interaction and other aspects.

## Additional file

Additional file 1 Dataset. Dataset of 194 human CaM-binding proteins and 193 Mitochondrial proteins used in this study. (PDF 36 kb)
