# Supplementary material for: The predictive performance of short-linear motif features in the prediction of calmodulin-binding proteins
Source: BMC Bioinformatics. 2018 Nov 20;19(Suppl 14):410. doi: 10.1186/s12859-018-2378-9 (PMC6245490; doi:10.1186/s12859-018-2378-9)
Supplement: Supplementary file 1 — Dataset. Dataset of 194 human CaM-binding proteins and 193 Mitochondrial proteins used in this study. (PDF 36 kb) [file 12859_2018_2378_MOESM1_ESM.pdf]

Table 1: Dataset of 194 human CaM-binding proteins and 193 Mitochondrial proteins used in this study.

194 CaM-binding Proteins									
O00222	O95716	P13797	P23634	P40145	P80723	Q13507	Q15506	Q99081	Q9UGI6
00329	P00533	P14151	P23763	P41594	Q00722	Q13554	Q15746	Q99250	Q9UIK4
O00418	P01236	P15735	P25098	P42336	Q01064	Q13576	Q15796	Q99578	Q9UL62
O00445	P01833	P15884	P27987	P42338	Q01668	Q13698	Q15797	Q99717	Q9ULV0
O00555	P02686	P15923	P28222	P43250	Q01814	Q13813	Q15835	Q99996	Q9UM54
O14830	P08034	P16520	P29033	P46939	Q01844	Q13884	Q15836	Q9H1R3	Q9UPR5
O14936	P08183	P17600	P29474	P46940	Q01970	Q13936	Q16566	Q9H2S1	Q9UQD0
O14994	P08237	P17677	P29475	P48454	Q02952	Q13972	Q16720	Q9H4G0	Q9UQL6
O15198	P08238	P17844	P29966	P48539	Q04206	Q14012	Q16816	Q9HAV0	Q9UQM7
O15554	P08908	P17858	P30301	P48736	Q04864	Q14028	Q60343	Q9HCX4	Q9Y210
O43525	P09496	P18433	P32298	P48995	Q05586	Q14123	Q86TI0	Q9NQ66	Q9Y2J2
O43526	P09497	P19634	P32418	P49006	Q05682	Q14524	Q86U12	Q9NQX4	Q9Y4H2
O43795	P11171	P20020	P34947	P49815	Q08209	Q14571	Q8NCM2	Q9NR82	Q9Y4I1
O43815	P11233	P20336	P35498	P53355	Q08828	Q14573	Q92686	Q9NRL3	Q9Y623
O75712	P11532	P20337	P35499	P54750	Q13033	Q14643	Q92736	Q9NYC9	
O94832	P12883	P20366	P35568	P55040	Q13255	Q14831	Q92777	Q9NZK7	
O95259	P13224	P21439	P35611	P55042	Q13402	Q14833	Q92952	Q9NZQ3	
O95377	P13533	P21579	P35612	P56696	Q13424	Q15051	Q92963	Q9UBC5	
O95452	P13535	P21817	P35626	P57103	Q13425	Q15075	Q96E17	Q9UBN4	
O95613	P13796	P23469	P38936	P61006	Q13459	Q15413	Q96RJ0	Q9UEY8	
193 Mitochondrial Proteins									
A8MTT3	O95167	P21549	P52789	Q15031	Q6NUN0	Q8IYU8	Q96DW6	Q9BXI2	Q9UGM6
A8MUP2	O95169	P21796	P53007	Q15119	Q6P1Q0	Q8IZJ6	Q96EL3	Q9BYT8	Q9UHN1
F7VJQ1	O95182	P21953	P55789	Q16540	Q6P587	Q8N0X4	Q96EY7	Q9BZ23	Q9UI43
O00217	O96008	P24310	P56385	Q16611	Q6UW78	Q8N159	Q96EY8	Q9BZE1	Q9UJS0
O00483	P00367	P30042	P59942	Q16740	Q6UXV4	Q8N357	Q96GC5	Q9H2K0	Q9UL12
O15091	P03891	P30049	P61604	Q16795	Q6ZSR3	Q8N3J5	Q96I99	Q9H2V7	Q9Y375
O15235	P03928	P30536	P78540	Q16798	Q6ZT89	Q8N442	Q96IL0	Q9H4I9	Q9Y3A0
O15528	P07954	P31415	P82673	Q16822	Q709F0	Q8N983	Q96NB2	Q9H5Q4	Q9Y3D6
O43181	P08574	P36776	P82909	Q16836	Q7KZN9	Q8NAN2	Q96RP9	Q9H7H0	Q9Y4W6
O43615	P09622	P36957	P82912	Q32NB8	Q7L4E1	Q8NB90	Q96T66	Q9HCM9	Q9Y5J7
O43715	P09669	P46199	P82933	Q5EBM0	Q7Z2W9	Q8TCC3	Q96TA2	Q9HD33	Q9Y5J9
O43716	P10176	P47985	Q02127	Q5HYI7	Q7Z6M4	Q8WUT9	Q99551	Q9NPB1	Q9Y5Z4
O60313	P11498	P48201	Q02318	Q5JRX3	Q7Z7H8	Q8WY22	Q99798	Q9NQH7	Q9Y6N1
O75027	P12694	P48735	Q08AH3	Q5RI15	Q86U28	Q92934	Q9BPX6	Q9NUJ1	
O75324	P13073	P49753	Q13011	Q5SXM8	Q86WU2	Q969S9	Q9BRQ8	Q9NVA1	
O75380	P13196	P49901	Q13114	Q5T653	Q86X76	Q96AG3	Q9BSK2	Q9NVH6	
O75390	P13995	P50213	Q13405	Q5TC12	Q86XF0	Q96C03	Q9BUE6	Q9NVS2	
O75394	P14317	P50336	Q13423	Q5TEU4	Q8IVH4	Q96D53	Q9BUK6	Q9NWU1	
O94925	P19099	P51687	Q13637	Q63ZY3	Q8IVP5	Q96DP5	Q9BW91	Q9NYZ2	
O95139	P20674	P51970	Q14410	Q6IB77	Q8IW45	Q96DV4	Q9BW92	Q9UGC7	
